# Supercritical Carbon Dioxide Technology for Recovering Valuable Phytochemicals from *Cannabis sativa* L. and Valorization of Its Biomass for Food Applications

**DOI:** 10.3390/molecules28093849

**Published:** 2023-05-01

**Authors:** Ana Carolina de Aguiar, Renata Vardanega, Juliane Viganó, Eric Keven Silva

**Affiliations:** 1 Centro de Ciências da Natureza, Universidade Federal de São Carlos, Rod. Lauri Simões de Barros, km 12–SP 189, Buri 18290-000, SP, Brazil; 2 School of Food Engineering, University of Campinas, Campinas 13083-970, SP, Brazil; 3 Centre of Biological Engineering, University of Minho, 4710-057 Braga, Portugal

**Keywords:** cannabidiol, THC, hemp seed bioproducts, high-pressure CO_2_

## Abstract

Supercritical carbon dioxide (CO_2_) extraction techniques meet all-new consumer market demands for health-promoting phytochemical compound-rich extracts produced from green and sustainable technology. In this regard, this review is dedicated to discussing is the promise of integrating high-pressure CO_2_ technologies into the *Cannabis sativa* L. processing chain to valorize its valuable pharmaceutical properties and food biomass. To do this, the cannabis plant, cannabinoids, and endocannabinoid system were reviewed to understand their therapeutic and side effects. The supercritical fluid extraction (SFE) technique was presented as a smart alternative to producing cannabis bioproducts. The impact of SFE operating conditions on cannabis compound extraction was examined for aerial parts (inflorescences, stems, and leaves), seeds, and byproducts. Furthermore, the opportunities of using non-thermal supercritical CO_2_ processing on cannabis biomass were addressed for industrial hemp valorization, focusing on its biorefinery to simultaneously produce cannabidiol and new ingredients for food applications as plant-based products.

## 1. Introduction

Supercritical fluid extraction (SFE) technology using CO_2_ as a solvent consolidated a special place among innovative solid-liquid extraction techniques used for recovering phytochemical compounds from plant matrices. Supercritical CO_2_ extraction processes meet modern consumer market demands for health-promoting bioactive compound-rich extracts, and also cover green chemistry concepts and sustainability principles. For this reason, SFE has been widely investigated as a smart strategy to integrate successful biorefineries involving valuable molecules applied as drug and therapeutic compounds.

Simultaneously with SFE technology, the scientific community’s interest to explore the *Cannabis sativa* L. as a source of therapeutic compounds for treating several disorders and diseases has grown greatly in recent years. Cannabis has emerged as the most investigated plant matrix for various purposes, including new drugs, cosmetics, herbal remedies, biofuel, food, building materials, paper, and others. It was promoted from a commonly used recreational drug to a promising natural drug for many health and well-being interests.

**Figure 1:**
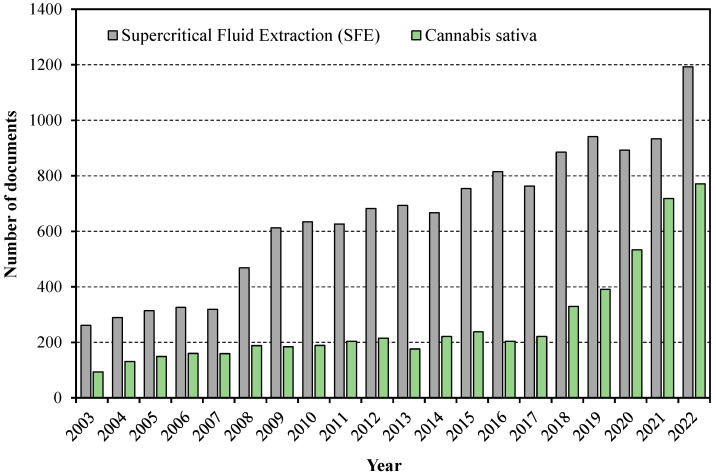
Number of documents (article and review) in Scopus database published from 2003 to 2022 using the keywords “supercritical fluid extraction” and “*Cannabis sativa*”.

Figure 1 exhibits the results returned for a search in the Scopus database using the keywords “supercritical fluid extraction” and “*Cannabis sativa*” in the last 20 years (from 2003 to 2022). The number of studies on both subjects has grown over the years. Comparing the first and last year, the number of scientific studies regarding SFE increased 4.57-fold while cannabis increased 8.20-fold. However, there was a strong growth in the dissemination of research about cannabis from 2017 to 2022. This almost exponential growth has placed cannabis as a hot research topic.

In this context, this review aimed to investigate the challenges and opportunities of integrating SFE technology into the cannabis processing chain for producing high-value-added extracts and new ingredients for food applications as plant-based products. To do this, the impact of SFE operating conditions on cannabis compound extraction was examined for inflorescences, stems, leaves, seeds, and byproducts. Furthermore, the therapeutic and side effects of cannabis were addressed.

## 2. Cannabis Plant, Cannabinoids and Endocannabinoid System

The *Cannabis* genus plants belong to the Cannabaceae family, from which the main species are *Cannabis sativa* and *Cannabis indica*. To date, more than 110 phytocannabinoids have already been identified in the *Cannabis sativa* L. plant, but Δ^9^-tetrahydrocannabinol (THC) and cannabidiol (CBD) are the most abundant, and consequently, are the most studied phytocannabinoids [1]. In Cannabis plants, the cannabinoids are present in its carboxylic forms Δ^9^-tetrahydrocannabinol acid (THCA) and cannabidiolic acid (CBDA). These compounds are synthetized from the same precursor, olivetoic acid, within the glandular trichomes present mainly in flowers of female plants. The genetic profile and relative level of expression of the enzymes responsible for the synthesis of THCA or CBDA (genotype), namely THCA synthase and CBDA synthase, respectively, determine the chemical composition of a particular cultivar (chemotype). Cannabis plants are mainly grouped into three chemotypes based on the absolute and relative concentrations of THCA and CBDA (Table 1), which enables distinguishing among the THC-type, also called the drug-type; and the CBD-type, mainly used for fiber production (industrial hemp) and present THC levels below 0.3% [2].

Currently, there are a plethora of substances generically called as cannabinoids which present effects associated with the cannabinoids’ receptors. The exogenous cannabinoids that are found in *Cannabis* plants are known as phytocannabinoids. The cannabinoids that are endogenously synthetized by physiological stimulation in humans are named endocannabinoids. Finally, there are also cannabinoids artificially synthetized which are called synthetic cannabinoids [3].

THC presents psychotropic effects, being responsible for the adverse psychedelic effects of cannabis, while CBD not only lacks these side effects, but can also module the THC activity, thus reducing its psychedelic effects [3,4]. Moreover, numerous other compounds have been found in cannabis, including terpenes, hydrocarbons, nitrogen-containing compounds, flavonoids, and phenolic compounds [5]. Despite the phytocannabinoids being described as mainly responsible for the therapeutic properties of cannabis, studies have demonstrated that the ingestion of terpenes like α-pinene, myrcene, limonene, and β-caryophyllene together with the cannabinoids can modulate its medicinal effects, which is called the entourage effect [6]. However, there is still no consensus about the existence of the entourage effect, since to date, no clear interaction pathway between cannabinoids and terpenes has been identified [7,8,9].

Currently, two cannabinoid receptors (CB) have been identified: CB1 and CB2, which belong to class A of the G-protein coupled family of receptors. CB1 receptors are mainly expressed in the central nervous system, being frequently found in the nerve cells of the amygdala, cortex, basal ganglia, hippocampus, and cerebellum [1]. On the other hand, CB2 receptors’ expression is more limited to immune system cells circulating through the bloodstream [10]. The CB1 and CB2 receptors, along with the endocannabinoids—mainly anandamide (AEA), 2-arachidonoylglycol (2-AG), and the enzymes responsible for their biosynthesis, transport, and degradation (such as fatty acid amide hydroxylase and monoacylglycerol)—comprise the endocannabinoid system (ECS) [11]. The ECS is an important and versatile physiological system involved in some of the main functions of the human body acting as a broad-spectrum modulator [10]. 

As ECS is involved in several human functions, including brain plasticity, learning, memory, neuronal development, cellular fate, nociception, inflammation, appetite regulation, digestion, suckling in the newborn, metabolism, energy balance, thermogenesis, motility, sleep-wake cycle, regulation of stress and emotions, and addiction [10], it has been suggested as an emerging target of pharmacotherapy, with therapeutic potential in almost all diseases affecting humans [10,12]. 

The exogenous cannabinoids (phytocannabinoids and synthetics) are all high-affinity agonists for both CB1 and CB2 receptors, which is one of the reasons why cannabis and their compounds and products are gaining paramount attention for the treatment of several diseases [13]. However, the high complexity of the ECS and species-specific characteristics has led to contradictory findings in preclinical studies, which is evidence that the mechanisms and actions of the ECS related to the therapeutic effects of exogenous cannabinoids are not completely elucidated, and are still the subject of research for scientists worldwide [13,14]. In addition, it is important to keep in mind that, although the phytocannabinoids are the major active compounds present in cannabis and in its preparations, the plant is not exclusively composed of substances that act on the CB1 receptors; the plant includes steroids, flavonoids, alkaloids, terpenes, among others, which are also the focus in intense pharmacological research [3,6].

## 3. Therapeutic Effects of Cannabis

There is a huge diversity of cannabinoid compounds being investigated for several potential therapeutic applications, but most of the findings are still considered of limited or insufficient scientific evidence to support the conclusion that cannabis or cannabinoids are an effective or ineffective treatment for the health endpoint of interest [2]. The therapeutic effects of phytocannabinoids, mainly THC and CBD, have already been extensively reviewed for application in the treatment of several diseases [15,16,17,18,19,20,21,22].

The fast-growing amount of scientific evidence regarding the positive effects of Cannabis-related products for the treatment of several diseases has supported the decriminalization of the cannabis possession and its legalization for medical treatments in several countries. In spite of promising developments in some Asian countries, such as the Philippines and Thailand, most Asian countries still maintain strict drug polices [13]. On the other hand, several Western countries have been taking place in the new cannabis market, with the commercialization of not only medical cannabis products, but other goods including vapes and edibles such as beverages and candies, for example [13,23].

In 2016, Alexander [16] reviewed the disorders in which cannabinoid ligands have clinical potential, including pain, nausea, vomiting, feeding disorders, glaucoma, neurodegeneration/neuroprotection, multiple sclerosis, schizophrenia, cancer, epilepsy, stress, and anxiety, concluding that the therapeutic areas best associated with exploitation of *Cannabis*-related medicines are pain, epilepsy, feeding disorders, multiple sclerosis and glaucoma.

The National Academies of Sciences, Engineering and Medicine (NASEM) conducted, in 2017 [2], a systematic and comprehensive review of over 10,000 abstracts of the recent medical literature on the health effects of cannabis and cannabinoids that enabled them to develop a standard language to classify the therapeutic effect of cannabis into four categories: 1. Conclusive evidence, 2. Moderate evidence, 3. Limited evidence, and 4. No or insufficient evidence to support therapeutic association [2]. The report concluded that there was conclusive or substantial evidence that Cannabis and cannabinoids are effective for the treatment of pain in adults, chemotherapy-induced nausea and vomiting, and spasticity associated with multiple sclerosis. Moderate evidence was found for secondary sleep disturbances. Limited, insufficient or absent evidence was reported for improvement in appetite, anxiety, Tourette syndrome, post-traumatic stress disorder, cancer, irritable bowel syndrome, epilepsy, and a variety of neurodegenerative disorders [2,15].

Recently, Fraguas-Sánchez and Torres-Suárez [1] published a chapter describing the current state of the therapeutic uses of *Cannabis sativa,* classifying the medical uses for multiple sclerosis, epilepsy, nausea and vomiting, pain, and appetite stimulation as high quality evidence, while the evidence for use in neurodegenerative disorders, cancer diseases, psychiatric disorders, alcoholism, and skin disorders as moderate–low quality.

All the aforementioned literature reports support that there is substantial evidence of the therapeutic effects of cannabis to treat pain, multiple sclerosis, and nausea and vomiting. Chronic pain is one of the most frequent reasons why patients are accessing medicinal cannabis [15,16,24]. The analgesic properties of THC has been investigated since this compounds was synthetized and evaluated for the first time in 1941 by Gaoni and Mechoulam [25]. Several studies have already demonstrated that ECS is expressed in the areas responsible for pain control; indeed, endocannabinoids are considered pain modulators, exhibiting an analgesic effect in both inflammatory and neuropathic pain models [1,16]. The majority of the studies implicate the CB1 receptor in the analgesic effects of cannabinoids, but there is also good evidence that CB2 receptor also contributes to these effects [16]. For more details about the mechanisms of action and results of the clinical trials that support pain control effects of Cannabis, the reader is encouraged to see Fraguas-Sánchez and Torres-Suárez [1], Guindon and Hohmann [26], Hutchison, et al. [27], Whiting et al. [22], and National Academies of Sciences, Medicine, Health, Medicine, Board on Population, Public Health, Committee on the Health Effects of Marijuana: An Evidence and Research [2].

Studies demonstrate that the use of medicinal cannabis for chronic pain can possibly reduce the use of opioids and other medications, which are associated with significant adverse effects, including dependence, risk of overdose, and gastric ulceration [27]. However, the challenge for cannabis-related medicines is to produce agents able to relieve the pain without intolerable adverse effects [16], since THC is also responsible for side effects due to the use medical cannabis, which will be further discussed in Section 4.

Multiple sclerosis is probably one disorder where cannabis has demonstrated to be highly effective [1]. Studies have demonstrated that in patients affected by multiple sclerosis, the expression of ECS is altered, and this alteration is related to the progression of the disease [28,29,30]. For example, CB2 receptors are only overexpressed in patients with primary-progressive disease [30]. ECS may be involved in multiple sclerosis physiopathology, explaining why cannabis is useful in the treatment of multiple sclerosis-related spasticity, a symptom that appears in 80% of patients [1,31]. To date, there is a licensed oral spray called Sativex^®^ that contains approximately 1:1 THC:CBD, and is recommended for the treatment of spasticity in adult multiple sclerosis patients, particularly in cases where other treatments fail [31]. The availability of licensed cannabis-based products to treat multiple sclerosis symptoms represent the result of substantial evidence that cannabis is effective as treatment [8].

The antiemetic properties of cannabis were firstly reported in 1975 by Sallan et al. [32], who observed considerable reducing in vomiting and nausea in patients receiving chemotherapy who smoked cannabis. Since then, the role of ECS in vomiting control has been extensively investigated, and in 1985, two synthetic analogues of THC, dronabinol and nabilone, were both approved for use in treating nausea and vomiting associated with cytotoxic chemotherapy [15]. Currently, there are at least four cannabinoids-based formulations containing dronabinol (Marinol and Syndros) and nabilone (Cesamet and Canemes) approved by FDA and EMA for use in treating chemotherapy-induced nausea and vomiting [1], but their commercialization is limited to only some countries worldwide.

Despite the antiemetic effectiveness of cannabinoids-based products in cancer patients mostly attributed to THC, recent studies have continued to evaluate the effects of cannabis extracts rich in both THC and CBD for this purpose, and showed that extracts containing THC and CBD at a ratio 1:1 reduced the nausea and vomiting in cancer patients receiving chemotherapy when administered thrice per day as add-on therapy [33]. The use of the combination between THC and CBD can be beneficial in terms of toxicity, as CBD reduces THC’s psychedelic effects [1]. 

National Academies of Sciences, Medicine, Health, Medicine, Board on Population, Public Health, Committee on the Health Effects of Marijuana: An Evidence and Research [2] considered that the evidence to support effects of cannabis in epilepsy is insufficient, because although all the studies included in the review reported benefit of the cannabinoid preparations used, the lack of blinding and control groups were deemed to make the evidence insufficient to support a benefit for cannabinoids in the treatment of seizures at this time [15]. However, other studies consider that there is substantial evidence to support that cannabis is effective in treating epilepsy [1,8,16,20].

The lack of effective medications to treat intractable forms of epilepsy, together with the anecdotal descriptions of therapeutic effects of CBD in these treatment-resistant epilepsies, specially Dravet syndrome, raised a fruitful future for CBD-related medicines in this indication, with preclinical evidence supporting the anticonvulsive properties of CBD [34,35]. Pamplona, da Silva, and Coan [20] conducted a meta-analysis with 670 patients describing the analysis of observational clinical studies on the treatment of refractory epilepsy with CBD-based products, aiming at attempting to establish the safety and efficacy of such products, in addition to the investigation of the existence of evidence to assume differences in efficacy between CBD-rich extracts and purified CBD products. The study concluded that treatments using CBD were effective and safe for the evaluated population with refractory epilepsy. The study also suggested that CBD-rich extracts are more potent than isolated CBD, since the administered average doses of CBD-rich extracts were lower (6.0 mg/kg/day vs. 25.3 mg/kg/day). In addition, mild to severe adverse events were more frequent in patients using purified CBD. The authors attribute the therapeutic advantages of CBD-rich extracts to the entourage effect, i.e., the synergistic effects of CBD with other phytocompounds present in the extract, but it remains to be confirmed in controlled clinical studies.

## 4. Side Effects

Despite all the therapeutic benefits of cannabis in several disorders, cannabinoids are also reported for presenting adverse effects in both medicinal patients and recreational users. The consumption of cannabis can initially produce euphoria and relaxation, and may also induce hallucinations, depression, and psychosis [36]. Other reported side effects include respiratory and cardiovascular disorders, cognitive alterations, and mood disorders [37]. The psychedelic side effects are mainly attributed to THC, since the consumption of pure CBD or CBD-rich preparations with insignificant THC levels do not present these effects [1]. Other common side effects associated with cannabis are dizziness, constipation or diarrhea, sedation, dry mouth, drowsiness, somnolence, nausea, vertigo, headache, anxiety, and fatigue, all of them mostly often described as low or moderate severity [31,37]. Another aspect related to cannabis consumption is potential addiction, which is mainly related to the presence of THC. In this sense, CBD products (isolate or extracts) with no or insignificant THC content are safer [1]. The most common side effects reported for CBD are tiredness, diarrhea and chances of appetite [38], but CBD can still be considered as having better side effects profile than most other drugs [13].

## 5. Supercritical Fluid Extraction (SFE)

There is no doubt about the increased interest in using products derived from cannabis in different industrial segments, especially regarding the compounds that have proven biological effects. However, as a rule, the development of products that exploit these compounds’ bioactive or technological potential is based on separation processes classified as solid-liquid extraction, which is a critical processing step with a broad spectrum of exploitation [39].

For years, the choice of extraction technique from plant matrices was based on maximizing the target compounds’ concentration and extraction yield. These characteristics are generally achievable through conventional extractions, such as those using reflux of organic solvents, maceration, hydrodistillation, and cold pressing. However, the needs of modern industry, based on the requirements of an increasingly critical society, have emerged, including the reduced use of petroleum-derived solvents and the development of toxic chemical-free production chains through sustainable processes and with the least possible environmental impact. Currently, a variety of alternative extraction processes, considered environmentally friendly, are available and can be selected depending on the desired extract type. For instance, microwave-assisted extraction (MAE) has been employed to obtain cannabinoids, phenolic compounds, and essential oils from cannabis [40,41,42]; ultrasound-assisted extraction (UAE) was used to produce extracts from cannabis containing cannabinoids, flavonoids, anthocyanin, stilbenoids, lignans, and oil [43,44,45,46]. In addition, high-pressure technologies, such as pressurized liquid extraction (PLE) and SFE, also have been largely employed. PLE was used largely to obtain cannabinoids [47,48,49]; meanwhile, SFE is the most applied method to obtain oil [50,51,52,53,54], cannabinoids [55,56,57,58,59,60,61,62], volatile compounds [59,63,64], and phenolic compounds [46].

Searches for the topic cannabis plus the extraction method were done in the Web of Science database, and it was evident that SFE is the most studied extraction method to obtain cannabis extract; SFE presented 58 results, followed by 29 for UAE, 21 for MAE, and 14 for PLE. Indeed, SFE applied to cannabis overcame the laboratory boundary and has extraction plants in different countries. Therefore, which are the factors that make SFE the preferred extraction method? SFE has several advantages; the possibility of fractionating the extract is the main feature. First, it is important to mention that carbon dioxide (CO_2_) is the preferable solvent in SFE. Therefore, the modulation of process conditions, mainly temperature and pressure, allow the supercritical CO_2_ to solubilize different compounds [65], resulting in selectivity. The fractionation can be achieved in at least two ways; separation vessels in series can be employed after the extraction, operating with different temperatures and pressures, and allowing the precipitation of extract with different chemical profiles; or temperature and pressure can be adjusted during the extraction to achieve the solubilization of different molecules [66]. Based on this feature, the selection of temperature and pressure maximizes the extraction yield and concentration of target compounds; meanwhile, the extraction of unwanted co-extracted ones is minimized [67,68].

Moreover, another peculiar feature of supercritical CO_2_ extraction is the absence of any solvent residue in the final extract, since CO_2_ is ventilated when it reaches atmospheric conditions [67]. Such a feature is very positive for pharmaceutical, cosmetic, and food applications, and avoids additional costs for solvent evaporation. In addition, CO_2_ is non-toxic, non-flammable, non-costly, and presents a low critical temperature (31.1 °C) and moderate critical pressure (7.4 MPa) [69]. Supercritical CO_2_ extraction is classified as environmentally friendly technology; through thermodynamic operations, CO_2_ is recycled and reused in the extraction [68]. 

It is worth mentioning that the decarboxylation process impacts cannabinoids’ obtention by SFE, since neutral forms are more soluble in supercritical CO_2_, and the natural form occurring in the cannabis is CBDA and cannabigerol (CBG). The decarboxylation principle is based on a reaction that converts acids into phenols. Therefore, CBDA is changed to CBD, and cannabigerolic acid (CBGA) to CBG. Such a reaction also occurs in the psychotropic compounds; THCA is converted to THC [66]. The conversion rates are functions of process temperature and time. For example, Marzorati et al. [70] pulverized the dried and milled inflorescences in a cold oven and increased the temperature to 1 °C/min until it reached 100 °C for 6 h, followed by SFE. Interestingly, Fernández et al. [58] performed the decarboxylation into the extraction vessel at 120 °C for 45 min immediately before SFE. As a result, the extract concentration in CBD and THC from pre-heated samples was significantly superior. However, the processing time is a critical factor in the cost analysis of SFE [71] because it impacts the annually produced batches. Therefore, cost analysis for this new approach is very welcome.

The supercritical CO_2_ extraction principle is based on the contact of CO_2_ at supercritical conditions and the raw material, which is placed into an extraction vessel where the CO_2_ flows, solubilizing the extractable compounds. Depressurization is allowed after the extraction vessel, and can be done in a single stage or series separators, as explained before. The extract precipitates and the CO_2_ is recycled [65]. Recycling, essential for large-scale installations, comprises of some steps to return the CO_2_ to the extraction line, and includes the removal of non-precipitated compounds in the separators, cooling, and pumping [71]. The main process variables are temperature, pressure, the solvent-to-feed (S/F) mass ratio, raw material particle size and moisture, and percentage of co-solvent when employed [72].

Temperature and pressure are the most important variables since they impact the density of the solvent. Temperature also affects the vapor pressure of the solute. The temperature decrease and pressure increase leads to an increase in the solvent density, and consequently increases the solubility of a wide range of compounds and increases the extraction yield. On the other hand, the temperature increase raises the vapor pressure of the solute, increasing the solubility and the extraction yield [72].

The S/F also greatly impacts the extraction yield and extract concentration. The spent mass of solvent (S) is a function of solvent flow rate and processing time in semicontinuous operation, and F is the feed of raw material. It is strongly advised to express the SFE yields as a function of S/F instead of extraction time. The extraction yield has a kinetic behavior, which means the accumulative mass of extract increases with S/F [73]. Moreover, S/F can also affect the extract composition; in low S/F, the very soluble compounds are first extracted, increasing their concentration in the extract; as the S/F is increased, such compounds are being depleted from the raw material, giving rise to the extraction of other compounds, and therefore changes the chemical profile of the extract [74].

Regarding the raw material, particle size reduction increases the surface area and mass transfer. However, very small particles can lead to bed compaction that hinders mass transfer. Another possible consequence is the formation of preferential bed channeling that reduces the mass transfer and leads to heterogeneous extraction from bed material [75]. Extraction yield increases with decreasing raw material moisture; high moisture content reduces the contact between solute and solvent, decreasing the mass transfer [76]. Consequently, a drying step before SFE may be necessary for raw materials with a high moisture content [68]. The selection of the drying methods and operational conditions, such as temperature, depends on the characteristic of the raw material and desired products; for instance, if the aim is to produce a concentrated extract in a specific thermosensitive compound, processing conditions must avoid thermal degradation.

Although supercritical CO_2_ has several advantages, there are also drawbacks associated with its application. One such drawback is that SFE plants require a significant fixed capital investment. Additionally, since supercritical CO_2_ selectively dissolves non-polar substances, polar compounds have low solubility in supercritical CO_2_ [68,74,77]. Solubility issues can be overcome by using co-solvents, which are generally added to the supercritical CO_2_ to modify the polarity [77]. Water and ethanol are the most used due to being considered green solvents [78]. Therefore, the addition of a co-solvent can increase the extraction of solutes with low solubility in pure CO_2_. However, the evaporation of the solvent from the extract is necessary depending on the extract application [72].

## 6. Impact of SFE Operating Conditions on Cannabis Compounds Extraction 

### 6.1. Cannabis Aerial Parts (Inflorescences, Stems and Leaves)

The studies available in the literature that evaluated the influence of operational parameters on the SFE process mostly use inflorescences or a mixture of the aerial parts (inflorescences, stems, and leaves) of the cannabis plant to obtain bioactive compounds. Table 2 and Table 3 summarize the main results of supercritical CO_2_ and supercritical CO_2_ plus co-solvent (ethanol) extraction from cannabis aerial parts to obtain cannabinoids and terpenoids, respectively.

Many strategies for recovering compounds with supercritical fluids from cannabis were studied, such as batch extraction under fixed operational conditions [70,79,80,81], sequential extraction process under different operational conditions (mainly temperature and pressure) in the same batch [82], extraction followed by fractionation [58,62,83], the use of co-solvents (ethanol) [58,60,81,83,84,85], in addition to performing the decarboxylation process inside the pressure vessel before SFE [58]. The process strategy and operating conditions determine the characteristics of the extracts, such as the extraction yield, recovery of compounds, and extract’s purity and composition, in addition to being related to technological aspects, such as solvent consumption, process time, among others.

**Table 2:** Most relevant studies for the recovery of cannabinoids with supercritical CO_2_ and supercritical CO_2_ plus co-solvent from cannabis aerial parts.

Molecules	Strain/Cultivar	Plant Part	Operational Conditions ^1^	Extraction Yield ^1^	Ref.
CBDA THC THCA CBDV THCV CBG CBGA CBN CBC	Cannatonic	Flowers	*T* (°C): 35, 39 and 45 *P* (MPa): 15, 20 and 25 *t* (min): 30, 120 and 180 *F* (g): 0.5, 0.6 and 1.0 *PS* (mm): <2.7	EY (%): 15.20 CBD (μg/mL): 195.26 CBDA (μg/mL): 603.37 THC (μg/mL): 21.06 THCA (μg/mL): 11.92 CBDV (μg/mL): 1.13 THCV (μg/mL): 0.76 CBG (μg/mL): 1.20 CBGA (μg/mL): 2.75 CBN (μg/mL): 0.59 CBC (μg/mL): 6.11	[80]
CBD THC	Seized cannabis bar	-	*T* (°C): 40, 60 and 80 *P* (MPa): 18, 25 and 32 *t* (min): 60 *F* (g): 600 *PS* (mesh): <40 Co-solvent: 0, 8.3, 16.7% ethanol	EY (%): 2.46 CBD (%): 11.92 THC (%): 19.52	[81]
CBD THC	Cultivar Helena	Aerial parts	*T* (°C): 40, 50 and 60 *P* (MPa): 10, 20 and 30 *t* (min): 60, 120, 180 and 240 *F* (g): 40	EY (%): 1.15 CBD (mg/g extract): 163.11 THC (mg/g extract): 6.58	[55]
CBD CBN THC	*Cannabis sativa* L.	-	*T* (°C): 42 and 50 *P* (MPa): 19–20, 25 and 29–30 *t* (min): 60, 90, 110 and 120 *F* (g): 420, 480, 500, 750, 900 and 920 *PS* (mesh):14 and 20–40 Co-solvent: 0, 1, 2, 3 and 4% ethanol	EY (%): 0.93 CBD (*w/w* extract): 10.30% CBN (*w/w* extract): 26.75% Δ^9^-THC (*w/w* extract): 2.140%	[83]
CBDV THCV CBD CBG CBDA CBGA CBN THC CBC THCA	Strain 1 Strain 2	Flowers	*T* (°C): 37 *P* (MPa): 25 *t* (min): 180 *F* (g): 1 *PS* (mm): <2.7	Strains 1 and 2, respectively CBD (μg/mL): 92.23 and 252.72 CBDA (μg/mL): 282.50 and 21.05 THC (μg/mL): 9.48 and 197.50 THCA (μg/mL): 4.71 and 1.60	[79]
CBDA CBD CBN CBCA CBC CBG THCA THC THCVA THCV CBGA CBDVA CBDV	*Cannabis sativa* L.	Inflorescences	Decarboxylation: 120 °C/45 min *P* (MPa): 20 and 30 *T* (°C): 50, 60 and 70 *t* (min): 15 (static) *PS* (mm): 0.5 to 2 CO_2_ flow rate (L/min): 0.50 (NTP) Co-solvent: 0 and 10% ethanol (*w/w*) *F* (g): 3.7 to 5.1 *S* (L): 100 (NTP)	THCA (g/100 g extract): 35.2 THC (g/100 g extract): 40.7 CBDA (g/100 g extract): 0.15 CBN (g/100 g extract): 0.119 CBCA (g/100 g extract): 2.67 CBC (g/100 g extract): 0.61 CBGA (g/100 g extract): 0.32 CBG (g/100 g extract): 0.35 THCVA (g/100 g extract): 0.35 THCV (g/100 g extract): 0.41	[58]
CBD THC	Narlı strain Elnur strain Papatya strain Gökçeağaç strain	Female leaves	Decarboxylation: 140 °C/30 min *P* (MPa): 15 and 33 *T* (°C): 40 and 60 CO_2_ flow rate (g/min): 100 *t* (h): 2 *F* (g): 100 Co-solvent: 0 and 2% (wt%)	CBD (Papatya strain): 3.71% THC (Papatya strain): 90.82% CBD (Elnur strain): 3.29% THC (Elnur strain): 58.22% CBD (Narlı strain): 7.70%	[84]
CBD THC	Mixture of cannabis plants of varying genotype (CBD:THC ~1:1.5)	Biomass	Decarboxylation: 120 °C/2 h *P* (MPa): 15, 23.5 and 32 T (°C): 60 CO_2_ flow rate (g/mL): 40, 95 and 150 *F* (g): 1000 *t* (min): 240, 420 and 600	CBD: 8.48 mg/g db (151.7 mg/g extract) THC: 4.99 mg/g db (187.6 mg/g extract)	[86]
CBD THC	Cultivar Finola	Inflorescences	Decarboxylation: 100 °C/6 h *P* (MPa): 38 *T* (°C): 60 *PS* (μm): ~50 *F* (g): 18 CO_2_ flow rate (m^3^/h): 0.28 8 cicles—10 min maceration (static conditions) + 10 min (dynamic conditions)	Without decarboxylation CBD (% *w/w*): 2.22 on dry biomass (15.8 mg/g extract) With decarboxylation CBD (% *w/w*): 6.21 on dry biomass (50.02 mg/g extract) THC (% *w/w*): 0.370 on dry biomass (3.01 mg/g extract)	[70]
THC CBD CBDA CBG CBGA CBN	*Cannabis sativa* L. different cultivars	Flower buds	Decarboxylation: 150 °C *P* (MPa): 30 *T* (°C): 50 *PS*: ~2 mm *Fractionation* 1° separator (P, MPa): 9, 11 and 13 2° separator (P, MPa): 5 *S/F*: 25 *Effect of co-solvent* Co-solvent: 5% ethanol *S/F*: 25 *Effect of pressure* *S/F*: 20 *P* (MPa): 20, 50, 70, 100 and 130	EY (wt%): 5.8 to 12 Total cannabinoids recovery (%): 51 to 100 CBD (mg/g): 449	[85]
THC	*Cannabis sativa* L.	Inflorescences	*P* (MPa): 15, 24 and 33 *T* (°C): 40, 60 and 80 *PS* (mm): <0.5 t (h): 4 CO_2_ flow rate (kg/h): 0.55 *F* (g): 8 *S/F*: 275 Co-solvent: 0, 2 and 5% ethanol	THC (% dry sample): 6.06 (5.38 recovery)	[60]
CBD CBDA CBG CBN THCA THC	*Cannabis sativa* chemovars Cherry kush Pineapple kush Purple sour diesel Ripped Bubba Harlequin	Flower trim	*T* (°C): 43 *t* (h): 6 *P* (psi): 1850 (12.76 MPa)	*Cherry kush chemovar* CBDA (mg/g extract): 91.2 CBD (mg/g extract): 5.3 THCA (mg/g extract): 693.8 THC (mg/g extract): 1.6 CBN (mg/g extract): 1.5 CBG (mg/g extract): 0.0	[59]
CBDA THC THCA	Hash Berry Sour Alien OG White Widow Abusive OG	Inflorescences	*P* (MPa): 17, 24 and 34 *T* (K): 328 *F* (g): 500	EY (g extact/g feed): 0.185 CBDA (%): 2.92 THCA (%): 70.56 THC (%): 25.78	[62]

^1^ *T*: temperature, *P*: pressure, *t*: extraction time, *F*: feed, *S*: solvent, *S/F*: solvent/feed, *PS*: sample particle size and EY: extract yield. CBD: Cannabidiol; CBN: Cannabinol; CBDA: Cannabidiolic acid; CBCA: Cannabichromenic acid; THC: Δ^9^-tetrahydrocannabinol; CBC: Cannabichomere; CBG: Cannabigerol; CBGA: Cannabigerolic acid; THCA: Δ^9^-tetrahydrocannabinolic acid, CBDV: Cannabidivann; THCV: Tetrahydrocannabivarin; THCVA: Δ^9^-tetrahydrocannabivarinic acid and CBDVA: Cannabidivarinic acid.

**Table 3:** Most relevant studies for the recovery of terpenes with supercritical CO_2_ and supercritical CO_2_ plus co-solvent from cannabis aerial parts.

Molecules	Strain/Cultivar	Plant Part	Operational Conditions ^1^	Extraction Yield ^1^	Ref.
endo-Fenchol *trans*-Pinene hydrate ⍺-Bisabolol	*Cannabis sativa* L.	Inflorescences	Decarboxylation: 120 °C/45 min *P* (MPa): 20 and 30 *T* (°C): 50, 60 and 70 *t* (min): 15 (static) *PS* (mm): 0.5 to 2 CO_2_ flow rate (L/min): 0.50 (NTP) Co-solvent: 0 and 10% ethanol (*w/w*) *F* (g): 3.7 to 5.1 *S* (L): 100 (NTP)	endo-Fenchol (%): 14.7 *trans*-Pinene hydrate (%): 4.5 ⍺-Bisabolol (%): 25.7	[58]
α-Pinene β-Pinene β-Myrcene D-Limonene Linalool Fenchyl alcohol α-Terpineol β-Caryophyllene α-Humulene α-Bisabolol	*Cannabis sativa* chemovars Cherry kush Pineapple kush Purple sour diesel Ripped Bubba Harlequin	Flower trim	*T* (°C): 43 *t* (h): 6 *P* (psi): 1850 (12.76 MPa)	*Cherry kush chemovar* α-Pinene (mg/g extract): 0.48 β-Pinene (mg/g extract): 0.48 β-Myrcene (mg/g extract): 0.13 D-Limonene (mg/g extract): 0.19 Linalool (mg/g extract): 3.14 Fenchyl alcohol (mg/g extract): 4.04 α-Terpineol (mg/g extract): 5.06 β-caryophyllene (mg/g extract): 20.60 α-Humulene (mg/g extract): 5.69 α-Bisabolol (mg/g extract): 4.53	[59]
α-Pinene Camphene β-Pinene Myrcene Limonene 1,8-cineol (Z)-ocimene (E)-ocimene γ-terpinene Terpinolene Linalool Caryophyllen (E)-b-farnesene α-Humulene Caryophyllene oxide β-Eudesmol β-Bisabolol α-Bisabolol	*Cannabis sativa* L.	Inflorescences	*F* (g): 150 *P* (MPa): 10 and 14 *T* (°C): 40 CO_2_ flow rate (kg/h): 3 *S/F*: 80 *Fractionation* *(2 separators: S1 and S2)* S1: 7 MPa and 25 °C S2: 5 MPa and 15 °C	%—peak area percentage α-Pinene (%): 13.78 Camphene (%): 0.53 β-Pinene (%): 4.23 Myrcene (%): 22.65 Limonene (%): 0.87 1,8-cineol (%): 0.80 (Z)-ocimene (%): 0.52 (E)-ocimene (%): 1.47 γ-terpinene (%): 0.62 Terpinolene (%): 7.55 Linalool (%): 1.91 Caryophyllen (%): 39.6 (E)-b-farnesene (%): 1.77 α-Humulene (%): 9.52 Caryophyllene oxide (%): 6.11 β-Eudesmol (%): 2.39 β-Bisabolol (%): 2.80 α-Bisabolol (%): 1.47	[63]

^1^ *T*: temperature, *P*: pressure, *t*: extraction time, *F*: feed, *S/F*: solvent/feed, *PS*: sample particle size and EY: extract yield.

As discussed earlier (see Section 5), pressure and temperature are the most critical variables in the SFE process. As the extraction yield of a specific compound by SFE depends on its solubility—which in turn is a function of the operating parameters—the process conditions are usually determined based on laboratory-scale experiments. For example, the solubility of CBD in supercritical CO_2_ is near to that of CBN (highest solubility at medium temperature, 50 °C), while CBG shows similarities with THC (highest solubility at high temperature, 70 °C). The differences in solubility of CBG, THC, CBD, and CBN are related mainly to their chemical structure and melting point [82].

Considering their chemical structure, CBN has the most aromatic character (6 double bonds), while Δ^9^-THC has the least aromatic character (4 double bonds). CBG and CBD present an aromatic character in-between (5 double bonds). As the CO_2_ interacts with the double bonds of the cannabinoids, a more aromatic character results in higher CO_2_ solubility. Cannabinoids’ melting point also influences their solubility in supercritical CO_2_. Liquid cannabinoids (CBD and CBN at 334 K and Δ9-THC at all temperatures) show lower solubility in supercritical CO_2_ compared to the solid cannabinoids (CBD and CBN at lower temperatures and CBG at all temperatures) [82].

In the literature, the experimental conditions evaluated for the recovery of cannabinoids and terpenoids are in the range of 10 to 38 MPa and 35 to 80 °C for operating pressure and temperature, respectively (Table 3 and Table 4). In this section, we propose evaluatingthe influence of the operational parameters on the extraction of cannabinoids and terpenoids by SFE from aerial parts of cannabis.

Regarding the operation pressure, at a fixed temperature, there is generally an increase in the recovery of cannabinoids (mass of an specific cannabidinol/mass of sample) with increasing pressure. Pressure positively influences the recovery of cannabinoids [62,85], although lower selectivities (mass of an specific cannabidinol/mass of extract) were recorded. Drinić, Vladic, Koren, Zeremski, Stojanov, Tomić and Vidović [55] found that an increase in pressure decreased the content of cannabinoids in the SFE extracts from aerial parts of *Cannabis sativa* (cultivar Helena). Although the highest extraction yield was obtained at the highest value of the tested pressure and temperature (30 MPa and 60 °C), the cannabinoid content in the extract was not the highest among the conditions studied. The low selectivity at high pressures observed by the authors was due to the increase in the solvation power of supercritical CO_2_ by increasing the pressure. Additionally, the costs of the pressurization step must be carefully evaluated in the definition of operational parameters on an industrial scale.

As previously mentioned, published data indicate that the solubility of different cannabinoids in SFE with supercritical CO_2_ varies with temperature. For example, increasing temperature results in an increase in the solubility of CBG and THC, and a decrease in the solubility of CBD and CBN [82]. Karğılı and Aytaç [84], when studying the SFE of cannabinoids from female cannabis leaves, found that at pressures lower than 15 MPa, the solubility decreases with the increasing temperature, while at pressures higher than 15 MPa, there was a reverse tendency. Based on the observed crossover region, the authors suggested that, at pressures below 15 MPa, the density effect is dominant; above this condition, the solute vapor pressure is the leading mechanism affecting the extraction process, while the volatility effect is dominant at higher pressures (> 15 MPa). Similar results were observed by Drinić et al. [55], which found that an increase in the temperature up to 60 °C at all investigated values of pressures (10, 20 and 30 MPa) had a negative effect on the cannabinoid isolation, except for THC at 30 MPa, where the increase in temperature had a positive influence.

The effect of other important operational parameters in SFE have also been evaluated. Co-solvents (mainly ethanol) were usually employed during SFE up to 16.7% (*w/w*) in relation to total CO_2_ (Table 2), and significantly increases cannabinoids extraction yield. Fernández et al. [58] concluded that the use of ethanol as a co-solvent during SFE significantly contributed to the extraction yield and cannabinoid recovery from cannabis inflorescences. At 70 °C e 30 MPa, the THC total recovery (defined as a percentage of total THC in the extract respect to plant material) increased from 75.0 to 92.2% with the addition of 10% ethanol as co-solvent. Moreno et al. [85] observed that CO_2_ plus 5% ethanol was more efficient than pure CO_2_ in extracting cannabinoid acids. In contrast, extraction using pure CO_2_ was very efficient for the extraction of neutral cannabinoids and reached a purity of about 45% in CBD at 20 MPa. Accordingly, Monton et al. [81], when optimizing the SFE process from seized cannabis bars, found that adding ethanol as a co-solvent (0 to 16.7%) did not promote an increase in the yield of cannabinoids (CBD and THC) extraction.

As observed, using ethanol as a co-solvent in the SFE process with supercritical CO_2_ is an interesting strategy to increase cannabinoid extraction yield, especially more polar compounds like acidic cannabinoids. However, it is important to highlight that adding co-solvents will require subsequent unit operations to remove it. Additionally, the use of co-solvents in SFE often implies a lower selectivity of the extraction process, resulting in extracts with higher concentrations of waxes and chlorophyll, which in turn requires more downstream processing for extract purification depending on the desired application [80].

Most studies have used time as an SFE process variable instead of evaluating the S/F parameter. As already highlighted in Section 5, it is strongly advised to express the SFE yields as a function of S/F instead of extraction time due to the kinetic behavior of the extraction yield. Qamar et al. [80], when evaluating the influence of extraction time on SFE optimization by fractional factorial design, found that the use of long extraction times (180 min) was a determining factor for higher cannabinoid extraction yields. Drinić et al. [55] evaluated the influence of the extraction time on the content of CBD and THC in the hemp extracts. The results indicated that over 70% of cannabinoids were extracted in the first 120 min of extraction. It is important to note that although it is possible to assess the influence of extraction time on SFE yield, more relevant information can be obtained from the point of view of process development by varying the S/F parameter. 

Although cannabinoids are the class of compounds of most significant interest when it comes to cannabis extraction, it is also important to assess the effect of SFE process conditions on the recovery of terpenoids present in cannabis plants. Da Porto et al. [63] performed supercritical CO_2_ extraction of terpenes on hemp inflorescences at a pressure of 10 and 14 MPa and a temperature of 40 °C. Online fractionation of the extracts was achieved by decreasing pressure and temperature in the two separators, S1 (7 MPa and 25 °C) and S2 (5 MPa and 15 °C). The extraction yield was significantly higher in S1 than in S2 for all extraction conditions. According to the results, cuticular waxes precipitated in S1 due to their lower solubility in supercritical CO_2_ compared to terpenes and their derivatives, while almost all volatile compounds were recovered in the S2 fraction. SFE at 10 MPa and 40 °C provided higher molecular weight compounds; namely, hydrocarbon sesquiterpenes (caryophyllene, β-farnesene, α-humulene) and oxygenated sesquiterpenes (caryophyllene oxide, β-eudesmol, β-bisabolol and α-bisabolol) were found in a lower percentage than at 14 MPa and 313.15 K. At constant temperature, the increase of pressure enhances the CO_2_ density, and consequently enhances its solvation power and the solubility of oxygenated sesquiterpenes in CO_2_. Therefore, the authors concluded that online fractionation was suitable for isolating hemp volatiles in the second separator.

### 6.2. Seeds

The SFE has been applied to cannabis seeds mainly to obtain oil (Table 4), whose extraction yields are very expressive; Aiello et al. [87] reported about 31 wt.% with a recovery of 93%, Devi and Khanam [52] obtained 36 wt.%, Tomita et al. [88] reached 44 wt.% that was equivalent to a recovery of 107.6%, and Da Porto et al. [50] achieved 22 wt.% with 72% of recovery. Aside from Tomita et al. [88], all works obtained these yields at 40 °C and 30–35 MPa. Tomita et al. [88] evaluated the effect of temperature (40, 60, and 80 °C) and pressure (20, 30, and 40 MPa) on the extraction yield. At 20 and 30 MPa, the temperature decrease led to higher yields. However, at 40 MPa, the increase in the temperature resulted in higher yields. Such an observation shows the crossover effect of density and solute vapor pressure. Regardless of the temperature, the increase in the pressure resulted in higher extraction yields.

**Table 4:** Most relevant studies for the recovery of compounds with supercritical CO_2_ from cannabis seeds and industrial residues.

Class of Compounds	Molecules	Strain/Cultivar	Plant Part	Operational Conditions^1^	Extraction yield ^1^	Ref.
Cannabinoids Polyphenols Tocopherols	Polyphenols α-tocopherol γ-tocopherol CBD CBN	*Cannabis sativa* L. USO31 cultivar	Seeds	*PS* ≤ 1 mm and 1 < *PS* < 2 mm *F* (g): 18 *T* (°C): 40 *P* (MPa): 30 CO_2_ flow rate (mL/min): 10 *t* (min): 195	EY (g/100 g): 30.98 ± 1.02 (93.19 ± 3.08% recovery) Polyphenols (GAE/kg oil): 51.42 ± 0.31 α-tocopherol (mg/kg oil): 39.57 ± 0.72 γ-tocopherol (mg/kg oil): 770.08 ± 10.75 CBD (mg/kg oil): 47.40 ± 0.85 CBN (mg/kg oil):76.52 ± 1.4	[87]
Fatty acids	Palmitic acid (C16:0) Stearic acid (C18:0) Oleic acid (C18:l) Linoleic acid (C18:2ω6) α-Linolenic acid (C18:3ω3)	*Cannabis sativa* L.	Seeds	*F* (g): 50 *t* (h): 4 *T* (K): 313.15, 333.15 and 353.15 *P* (MPa): 20, 27.5 and 35 CO_2_ flow rate (g/min): 5, 10 and 15 *PS* (mm): 0.430, 0.675 and 1.015 Co-solvent: 0, 5 and 10 % of CO_2_ flow rate	EY (%): 36.26 C16:0 (%): 2.52 C18:0 (%): 0.44 C18:l (%): 8.09 C18:2ω6 (%): 51.38 C18:3ω3 (%): 21.41	[52]
Fatty acids β-carotene Total tocopherols	Palmitic acid (C16:0) Palmitoleic acid (C16:1ω7) Stearic acid (C18:0) Oleic acid (C18:l ω9) Linoleic acid (C18:2ω6) α-Linolenic acid (C18:3ω3) C20:1ω9 C22:1ω9 β-carotene Total tocopherols	*Cannabis sativa* L.	Seeds	*T* (°C): 40 and 60 *P* (MPa): 30 and 40 CO_2_ flow rate (mL/min): 1.15 *F* (g): 4 *t* (min): 240	C16:0 (%): 6.28 C16:1ω7 (%): 0.10 C18:0 (%): 2.61 C18:1ω9 (%): 12.64 C18:2ω6 (%): 57.99 C18:3ω3 (%): 18.54 C20:1ω9 (%): 0.35 C22:1ω9 (%): 0.02 Total tocopherols (mg/L): 935.5 β-carotene (mg/L): 16.84	[89]
Tocopherols Fatty acids Pigments	α-tocopherol γ-tocopherol Total chlorophyll Total carotene Palmitic acid (C16:0) Oleic acid (C18:0) γ-Linolenic acid (C18:3ω6) α-Linolenic acid (C18:3ω3) Linoleic acid (C18:2ω6)	*Cannabis sativa* L. Genotype *Fedora 17*	Seeds	*F* (g): 100 g *P* (MPa): 30 and 40 *T* (°C): 40 and 60 CO_2_ flow rate (kg/h): 1.94	α-tocopherol: 189.08 mg/L γ-tocopherol: 134.06 mg/L Total chlorophyll: 90.65 mg/kg Total carotenoids: 34.21 mg/kg C16:0 (%): 6.92 C18:0 (%): 13.17 C18:3ω6 (%): 3.16 C18:3ω3 (%): 16.29 C18:2ω6 (%): 58.19	[90]
Fatty acids	Palmitic acid (C16:0) Stearic acid (C18:0) Oleic acid (C18:1) Linoleic acid (C18:2ω6) Linolenic acid (C18:3ω6)	*Cannabis sativa* L.	Seeds	*F* (g): 4 CO_2_ flow rate (mL/min): 3 *t* (min): 0–180 (kinetic experiments) *T* (°C): 40, 60 and 80 *P* (MPa): 20, 30 and 40	EY (%): 0.442 g/g sample C16:0 (%): ~10% C18:0 (%): ~3% C18:1 (%): ~10% C18:2ω6 (%): ~17% C18:3ω6 (%): ~60%	[88]
Fatty acids	Palmitic acid (C16:0) Stearic acid (C18:0) Oleic acid (C18:1) Linoleic acid (C18:2ω6) Linolenic acid (C18:3ω6) α-Linolenic acid (C18:3ω3) Eicosenoic acid (C20:1) Behenic acid (C22:0)	*Cannabis sativa* L.	Seeds	*T* (°C): 40, 50 and 60 *P* (MPa): 25, 30 and 35 *PS* (nm): 0.59, 0.71 and 0.83 CO_2_ flow rate (kg/s): 8 × 10^−5^ *t* (min): 60 *F* (g): 15 g	C16:0 (%): 5.85 ± 0.06 C18:0 (%): 1.45 ± 0.04 C18:1(%): 10.67 ± 0.14 C18:2ω6 (%): 59.21 ± 0.70 C18:3ω6 (%): 3.40 ± 0.09 C18:3ω3 (%): 18.47 ± 0.63 C20:1 (%): 0.12 ± 0.06 C22:0 (%): 0.84 ± 0.01	[51]
Fatty acids	Palmitic acid (C16:0) Stearic acid (C18:0) Oleic acid (C18:l) Linoleic acid (C18:2ω6) γ-Linolenic acid (C18:3ω6) α-Linolenic acid (C18:3ω3) Eicosenoic acid (C20:l) Behenic acid (C22:0)	*Cannabis sativa* L.	Seeds	*F* (g): 300 g CO_2_ flow rate (kg/h): 10 *T* (°C): 40, 60 and 80 *P* (MPa): 30 and 40 *S/F*: 30, 45 and 60 *PS*: 1.50 mm	EY (%): 22.1 ± 0.7 (72.2 ± 0.5% recovery) *Main fatty acids (mean value for different conditions)* C18:l (%): 11.25 C18:2ω6 (%): 59.47 C18:3ω3 (%): 18.08	[50]
Cannabinoids	CBD CBC THC CBG CBN CBDA THCA	*Cannabis sativa* L.	Industrial hemp threshing residue (stalks and leaves)	*F* (g): 500 CO_2_ flow rate (kg/h): 7 *T* (°C): 45 *P* (MPa): 10 and 45 2 Separators (S1 and S2) *T* (°C) S1 and S2: 45 *P* (MPa) S1: 8–9 *P* (MPa) S2: 4	CBD (mg/100 g db): 788.0 CBDA (mg/100 g db): 1660.9 CBC (mg/100 g db): 32.3 CBG (mg/100 g db): ~15 THC (mg/100 g db): 18.6 THCA (mg/100 g db): 46.1	[91]
Cannabinoids	CBD CBDA	*Cannabis sativa* L.	Threshing residue	*F* (g): 10 CO_2_ flow rate (L/min): 2–3 (at 0.0018 g/mL CO_2_ density) *P* (MPa): 10, 30 and 50 *T* (°C): 35, 52.5 and 70 *t* (min): 60, 90 and 120	EY (g/100 g dw): 10.36 ± 0.31 CBD: 0.23 ± 0.01 g/100 g dw (24.15 ± 0.89 mg/g extract) CBDA: 0.16 ± 0.00 g/100 g dw (239.3 ± 1.0 mg/g extract)	[92]
Cannabinoids Lipophilic compounds	CBD Fatty acids Policosanols Fatty aldehydes Hydrocarbons Sterols Triterpenoids	*Cannabis sativa* L.	Dust residues	*P* (MPa): 8, 24, 35 and 40 *T* (°C): 35, 50 and 65 *F* (g): 100 *t* (h): 4 CO_2_ flow rate (g/min): 35	Total fatty acids (μg/g of dust): 2252.8 ± 108.5	[93]

^1^*T*: temperature, *P*: pressure, *t*: extraction time, *F*: feed, *S/F*: solvent/feed, *PS*: sample particle size and EY: extract yield. CBN: Cannabinol; CBD: Cannabidiol; CBDA: Cannabidiolic acid; THCA: Δ^9^-tetrahydrocannabinolic acid; THC: Δ^9^-tetrahydrocannabinol; CBC: Cannabichomere and CBG: Cannabigerol.

In addition to the overall extraction yield, the works presented in Table 4 also show the fatty acids profile and the content of polyphenols, tocopherols, carotenoids, and cannabinoids in the oil. The primary fatty acids in the seed oil are linoleic (51–59%), linolenic (16–21%), oleic (8–12%), stearic (0.4–13%), and palmitic (5–10%). The content of polyphenols, CBD, and CBN was reported by Aiello et al. [87]; however, the concentration of such compounds is very low: 51, 47, and 76 mg/kg, respectively. Similarly, the β-carotene was reported by Grijó et al. [89] (17 mg/L), and total carotenoids by Aladić et al. [90] (34 mg/kg). Regarding the tocopherols, Aiello et al. [87] identified γ-tocopherol as the majority (770 mg/L), and Aladić et al. [90] identified the α-tocopherol (189 mg/L); however, the used strains by both works were different, which can lead to the divergent results.

### 6.3. By-Products

The use of agro- and food industrial byproducts or wastes has shown much progress in recent years. Industrial hemp is cultivated to produce fibers for paper and textile, cellulose, oil, cosmetics, and pharmaceuticals [92]. The harvesting and processing lose a considerable amount of threshing, containing substantial amounts of valuable compounds [91]. Threshing residue was investigated by Kitrytė et al. [92] and Vági et al. [91] (Table 4). Kitrytė et al. [92] proposed biorefining the threshing into cannabinoids and antioxidant fractions. Supercritical CO_2_ extraction was used to obtain the lipophilic fraction containing fatty acids and cannabinoids, recovering more than 93% of the cannabinoids from the raw material. The SFE residue was used as raw material in PLE to extract flavonoids, and the residue from PLE was applied to enzyme-assisted extraction to release mono- and disaccharides. The whole sequential process reduced the raw material by 90–99%. Vági et al. [91] obtained cannabinoids from threshing residue using supercritical CO_2_ extraction and verified the increase in the yield with pressure without a significant increase in the cannabinoids yield. Attard et al. [93] (Table 3) performed supercritical CO_2_ extraction on the dust from the fiber extraction, ranging temperature and pressure; the authors verified that conditions of 40 MPa and 65 °C produced the highest yield of crude wax, and at 35 MPa and 50 °C, the highest yields of fatty alcohols, fatty aldehydes, alkanes, sterols, and CDB were obtained.

## 7. SFE of Cannabis: Challenges and Prospects 

Although SFE is one of the most appropriate techniques for extracting cannabinoids, some challenges still need to be overcome to exploit the full potential of cannabis from an industrial perspective. Aspects related to the sustainability of the production chain, in particular the full use of raw material and reduction of solvent and energy consumption, in addition to the quality of the extracts, must be continually evaluated to guarantee adherence to the new demands that are imposed on industrial processes.

A strategy that has been gaining prominence in the development of SFE processes is the full use of the materials in a biorefinery or sequential process approach [65]. In this approach, in addition to obtaining compounds of interest by SFE under different operating conditions (temperature, pressure, co-solvent, etc.) during the same batch, it is possible to submit pre-SFE-extracted material to other extraction steps intending to recover different classes of compounds (e.g., phenolic compounds) by using emerging extraction techniques (PLE, UAE, enzyme-assisted extraction, etc.) [92]. It is also possible to carry out pre-treatments of the raw material, such as the decarboxylation process in situ immediately before SFE extraction without the presence of CO_2_ [58]. Speier [94] filed a patent application for a sequential SFE process on *Cannabis sativa* L., formed by up to four extractions in series, claiming these processes in an extensive range of temperatures (−15/200 °C) and pressures (5.2/172.3 MPa), without specifying the supercritical fluid employed.

Another relevant challenge regarding the extraction of bioactive compounds from cannabis by SFE is the production of single cannabinoids targeting applications that demand greater purity, such as pharmaceutical applications and in the application production of fortified foods [66]. As discussed earlier, the SFE process can produce extracts with a mixture of compounds, which can be more or less concentrated in a class of compounds depending on the operating conditions selected. For applications that require high-purity extracts in a particular class of compounds, adding further steps of fractionation with CO_2_, such as continuous countercurrent column and pressure reduction, has shown to be a promising alternative. Both fractionation processes are based on the solubility difference of the liquid mixture components in the supercritical solvent [95]. Based on this approach, Baskis [96] patented a complex multistep process involving a supercritical fluid step to isolate and purify the cannabinoid mixture obtained in the previous stages of the process into individual cannabinoids.

For applications that demand a high degree of purity of a single compound (≥ 99%)—for example in CBD and CBG for pharmaceutical applications—it can be useful to apply chromatographic techniques at an industrial scale. Preparative chromatography is based on the principle that different cannabinoids travel through a specific stationary phase at different speeds; consequently, compounds are separated and can be successively collected [97]. Among these chromatographic techniques, high-pressure flash chromatography, centrifugal partition chromatography, supercritical CO_2_ chromatography, and simulated moving bed chromatography appear as the most interesting alternatives [66,70].

Finally, developing refining processes for the extract obtained by SFE, such as the winterization step, but using supercritical CO_2_ instead of ethanol, is also a possibility that can be explored to increase the quality and sustainability of phytochemical cannabis products.

## 8. Non-Thermal Supercritical CO_2_ Processing of Cannabis Biomass 

**Figure 2:**
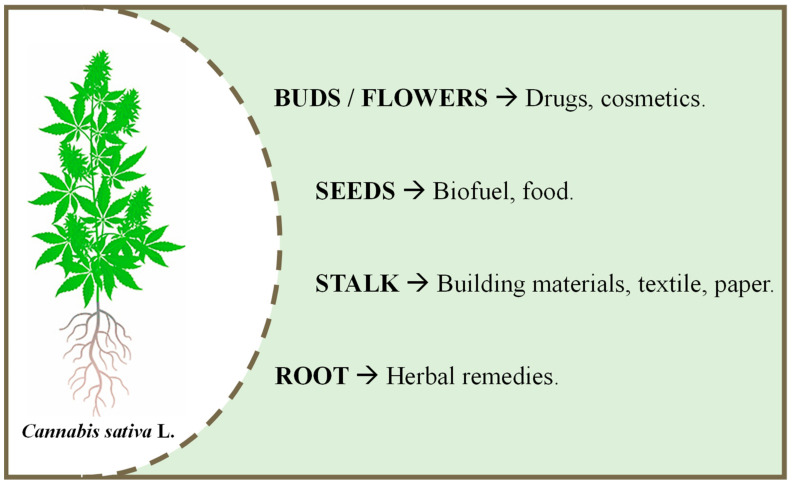
Industrial applications of *Cannabis sativa* L. related to each plant fraction (Adapted from Hesami et al. [109]).

Beyond the biorefining approach to enhance the cannabis SFE extracts by integrating innovative green technologies, supercritical CO_2_-based manufacturing processes have been highlighted due to the slight impact of their application on different macronutrients, such as proteins, sugars, starches, dietary fibers, and others [98,99,100,101,102]. *Cannabis sativa* L. exhibits many industrial properties for application in different sectors depending on its plant fraction. Figure 2 summarizes the potential uses of cannabis according to its plant fraction. Among hemp bioproducts, seeds have a high market potential in crucial industrial sectors, including food, pharmaceutical, chemical, and bioenergy [103]. Hemp seeds are recognized as a good source of fat, protein, fiber, minerals, and bioactive compounds, such as carotenoids, tocopherols, and sterols [104]. In this regard, cannabis biomass after supercritical CO_2_ processing may present technological properties and physicochemical characteristics similar to unprocessed raw material. Therefore, the non-thermal processing of cannabis biomass brings various opportunities for industrial hemp valorization focusing on its biorefinery to simultaneously produce CBD and bioproducts for food applications.

Thermal treatments are an important manufacturing step both for ensuring the safety of foods and beverages and extending their shelf-life. Heat application (60 °C–200 °C) is the most traditional preservation method used to inactivate pathogenic and spoilage microorganisms and endogenous enzymes. It includes various methods, such as pasteurization (high-temperature and short-time—HTST and low-temperature long-time—LTLT), sterilization (ultra-high temperature—UHT), cooking, steaming, roasting, boiling, and others [105,106]. However, severe thermal processing may promote undesirable changes on food compounds, such as negative effects on organoleptic, physical, chemical, and nutritional properties [98]. In this context, non-thermal emerging technologies have gained audience due to their performance retaining nutritional and sensory qualities in addition to maintaining the freshness of food products. Some studies have hypothesized that non-thermal treatments are often less detrimental to food matrices because they generally impact hydrophobic bonds, hydrogen, electrovalent bonds, and ionic bonds, i.e., non-covalent bonds. In contrast, thermal processing may affect non-covalent and covalent bonds [107,108].

As discussed before, CO_2_ presents a low critical temperature (31.1 °C) and moderate critical pressure (7.4 MPa). Thus, its critical point enables designing non-thermal processes with pressure conditions that are easier to achieve compared to other non-thermal high-pressure technologies, such as high-pressure processing (HPP), which requires pressure ranges from 50 to 1000 MPa [110]. On the other hand, CO_2_ comes into direct contact with the plant matrix during the extraction process, diffusing through plant tissues to extract compounds of interest. The key point that makes CO_2_ an attractive solvent for food intents is the fact that it is a substance that is not chemically reactive [111]. Indeed, many studies reported the chemical stability of macronutrients after supercritical CO_2_ processing such as sugars [99,102], dietary fibers [100], proteins [112,113], and others. 

Regarding proteins, supercritical CO_2_ treatment has been used to promote new opportunities for extending the application of plant proteins in food products by enhancing their technological properties. Sheikh, Saini and Sharma [112] reported that plum (*Prunus domestica* L) kernel protein isolate had its solubility and foam capacity improved after supercritical CO_2_ treatment at 20 MPa for 60 min with a temperature ranging from 30 to 70 °C. Supercritical CO_2_-treated samples had their solubility increased up to 23% and foam capacity up to 200% compared to the native plant protein. The improvements in the plant protein’s technological properties were attributed to the CO_2_ solubility that promoted significant changes in protein conformation, making them more prone to aggregation forming soluble aggregates [112]. Therefore, supercritical CO_2_ processing could be a smart strategy for the underutilized novel plant protein sources. For many years, the use of hemp seed proteins have had many restrictions in the food industry, mainly due to their technological properties. They exhibit poor solubility, emulsifying, and foaming properties in water [114]. 

**Figure 3:**
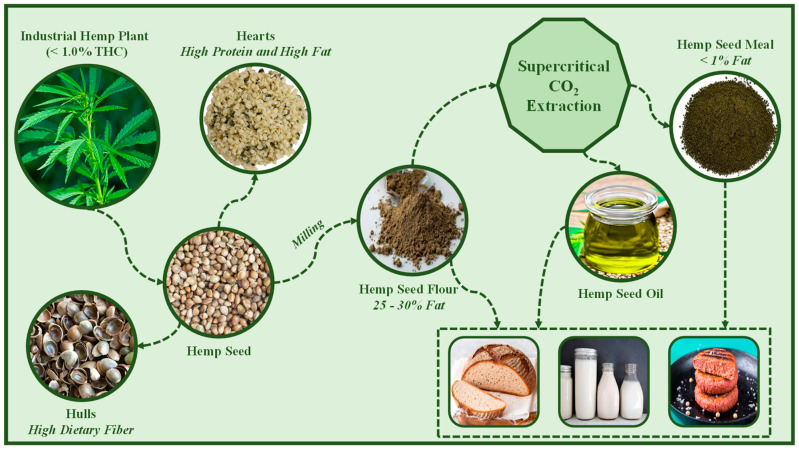
Food-grade ingredients and products from hemp seed obtained from supercritical CO_2_ extraction technique.

High-pressure CO_2_-based manufacturing processes are a promising non-thermal option to integrate biorefineries involving hemp seed biomass. Figure 3 illustrates high-value-added products obtained from a biorefinery dedicated to valorization of cannabis seeds from industrial hemp plants (<1.0% THC) employing supercritical CO_2_ as an extraction technique of hemp seed oil. Hemp seed flour is a good ingredient to improve the nutritional balance of plant-based foods. Rice-based yogurt was fortified with hemp seed flour to produce a new beverage with suitable nutritional, functional, and sensory attributes. The hemp seed flour addition contributed to the high protein, fiber, and mineral contents of the yogurt-like product, assisting the fermentation by selected lactic acid bacteria and decreasing the predicted glycemic index [115]. Nissen et al. [116] evaluated the hemp seed flour as a healthier novel matrix to produce plant-based alternative milk because it is an important source of nutrients, antioxidant compounds and bioactive molecules (polyunsaturated fatty acids). Furthermore, the hemp seed matrix boosted the production of acetate, propionate, and butyrate short-chain fatty acids during the fermentation process using probiotic strains. Likewise, Zahari et al. [117] developed a meat analogue from hemp seed protein concentrate to investigate the maximum ratio replacement of soy protein by hemp protein in the formulation of the meat substitute. They concluded that soy protein could be replaced by hemp protein by up to 60%. Rusu et al. [118] fortified wheat bread with hemp seed flour and obtained a new bakery product with a high content of proteins containing essential amino acids, as well as unsaturated fatty acids, fibers, and minerals, maintaining the rheological characteristics of the wheat bread.

All food-grade ingredients and products from hemp seed shown in Figure 2 meet the new global demands of the consumer market for natural products, are free of toxic solvents, and are produced from fresh ingredients with sensory attributes similar to the unprocessed product. In this way, hemp seed oil and meal could be used to fortify bakery products (breads, cakes, cookies), salad dressings, ice creams, and can be used to produce hemp-based milk and meat. Additionally, all food products were also produced from ingredients obtained through a green and sustainable process.

## Figures and Tables

**Table 1:** Cannabis phenotypes [2].

Chemotype	THCA (%)	CBDA (%)	CBDA/THCA Ratio
THC-type	0.5–15	0.01–0.16	<0.02
Hybrid	0.5–5	0.9–7.3	0.6–4
CBD-type	0.05–0.7	1.0–13.6	>5

## Data Availability

Not applicable.
